# Respiratory syncytial virus evaluation among asymptomatic and symptomatic subjects in a university hospital in Sao Paulo, Brazil, in the period of 2009‐2013

**DOI:** 10.1111/irv.12518

**Published:** 2018-02-04

**Authors:** Luciana Peniche Moreira, Aripuana Sakurada Aranha Watanabe, Clarice Neves Camargo, Thais Boim Melchior, Celso Granato, Nancy Bellei

**Affiliations:** ^1^ Medicine Department Sao Paulo Federal University Sao Paulo Brazil

**Keywords:** asymptomatic, respiratory syncytial virus, symptomatic, viral load

## Abstract

**Background:**

The respiratory syncytial virus (RSV) is recognized as an important cause of respiratory tract infections. Immunocompromised patients, healthcare workers (HCWs) and children contacts are at increased risk of acquiring the infection. However, the impact of asymptomatic infection in transmission has not been well studied. Objectives: this study evaluated the frequency and viral load (VL) of RSV in nasal swab samples of individuals with different risk factors for acquiring infection in a university hospital in Sao Paulo, Brazil.

**Methods:**

We included 196 symptomatic children and their 192 asymptomatic caregivers, 70 symptomatic and 95 asymptomatic HCWs, 43 samples from symptomatic HIV‐positive outpatients, and 100 samples of asymptomatic HIV patients in the period of 2009‐2013.

**Results:**

RSV infection was detected in 10.1% (70/696) of samples, 4.4% (17/387) of asymptomatic patients, and 17.1% (53/309) from symptomatic patients. (*P* < .0001). The VL of symptomatic patients (4.7 log copies/mL) was significantly higher compared to asymptomatic patients (2.3 log copies/mL). RSV detection among asymptomatic caregivers (6.8%; 13/192) was significantly higher compared to other asymptomatic adults, HIV and HCWs (2.0%; 4/195; *P* = .0252). A close contact with an infected child at home was an important risk to RSV acquisition [OR 22.6 (95% CI 4.8‐106.7)]. Children who possibly transmitted the virus to their asymptomatic contacts had significantly higher viral load than children who probably did not transmit (*P* < .0001).

**Conclusions:**

According to our results, it is important to know if people circulating inside the hospital have close contact with acute respiratory infected children.

## 1 INTRODUCTION

The respiratory syncytial virus (RSV) has great importance as a causative agent of nosocomial infections, especially for its particles being highly contagious.^1^

Respiratory syncytial virus severe clinical outcomes in children and immunocompromised patients are largely known; however, the studies including asymptomatic individuals with different levels of exposure to this pathogen in different sets are scarce.^2^, ^3^Asymptomatic respiratory viral infections occur in a variable frequency, and among young children, it can be high.^4^ The occurrence of transmission between children and their asymptomatic contacts is still insufficiently studied, despite their importance in the virus transmission chain. It is known that asymptomatic excretion of RSV occurs in 15%‐20% of the infected healthcare workers (HCWs).^1^ In other adults, the asymptomatic RSV infection is still barely studied and the researchers keep trying to understand the detection of genetic particles in their samples.^5^ For example, a few studies have been conducted regarding the frequency of RSV infection in patients HIV positive.^6^, ^7^

This study aims to evaluate the occurrence of symptomatic and asymptomatic infections caused by RSV by analyzing samples collected from patients, healthcare workers and companions of patients in a university hospital complex. The frequency of RSV detection in individuals with different risk factors for acquiring infection is described. Comparisons of viral load were also performed. The individuals included in this study were HCWs, HIV‐infected patients, children, and caregivers of children with respiratory symptoms.

## 2 METHODS

### 2.1 Study design

This was a cross‐sectional study in which patients were enrolled through active recruitment. A total sample size of 696 nasal swab samples was established considering the 6 groups studied: symptomatic and asymptomatic healthcare workers; symptomatic and asymptomatic HIV‐positive individuals; symptomatic children; asymptomatic caregivers of symptomatic children.

The sample size was calculated to be sufficient to determine a prevalence of RSV at least up to 10%, with 90% confidence interval and a relative precision of 0.25 of the a priori estimated proportion. The goal of sampling was that we could determine the prevalence of RSV in 4 different groups.

These samples were collected in the period of 2009‐2013, in different care units of a university hospital complex in Sao Paulo, Brazil, during all over the year, including RSV season and off‐season months. The university hospital has 700 beds and is a referral hospital with both basic services and specific care from patients attended at primary care health service in ambulatory or specific units.

### 2.2 Patients

The inclusion criteria for asymptomatic adults was the absence of any respiratory symptoms during the 15 days before sample collection; for symptomatic adults and children (up to 12 years of age), the criteria was the diagnosis of acute respiratory infection (ARI) within a week before sample collection. Acute respiratory infections were defined as fever or feverish and cough or sore throat. For the analysis among symptomatic children and their asymptomatic caregivers, the child was considered as “possibly transmitting” if had a positive sample and their related caregiver also had a positive sample.

### 2.3 Samples

All the volunteers were invited to participate and, after appropriate clarifications, signed a free informed consent. The study was approved by the UNIFESP Research Ethics Committee (Process number 369.760). The volunteers or their companion filled a questionnaire about the clinical signs, and the collected samples were immediately processed and stored. The healthcare workers (physicians and nurses who had direct contact with patients) were actively recruited in different hospital wards/clinics (Nephrology, Pediatrics, Haematology, Infectious Diseases, Transplant, Cardiology, Psychiatry, General Surgery, Laboratory And Intensive Care Unit) of the university hospital twice a week. The HIV‐positive patients were enrolled during the schedule routine visits once a week in the outpatient clinic of the Infectious Diseases Department. The symptomatic children and caregivers were enrolled, twice a week, in the pediatrics sector of the Employee Health Care Center (NASF) during medical care. One researcher interviewed a pair of asymptomatic caregivers and a symptomatic child, referred by a pediatrician, and samples were collected only from children which the asymptomatic caregiver agreed to participate.

### 2.4 Laboratory methods

Nasopharyngeal swabs were collected, immediately transported to the virology laboratory, and aliquots were frozen at −80°C for further analysis by PCR. Nucleic acid was extracted from 140 μL using the ”QIAmp Viral RNA Extraction Kit” (Qiagen, Hilden, Germany) according to the manufacturer's instructions.

### 2.5 RSV detection through qRT‐PCR

For the detection of RSV, we selected the protocol described by Homaira et al, 2012.^8^ We used primers and probe specific for the P gene of the virus. The reactions were performed in 96‐well plates, using AgPath IDTM One‐Step RT‐PCR kit (Ambion, Foster city, CA, USA) according to the manufacturer's instructions. The RNase P gene^9^ was used as the internal control and for the normalization of sample concentrations. The amplification and fragment detection were done on the 7500 Real Time PCR System (Applied Biosystems, Foster City—EUA, CA, USA). Samples viral loads were normalized according to the formula: normalized sample Ct = (Ct sample) x (Ct RNase P of the sample)/ average Ct value of all RNase P samples).^10^, ^11^

### 2.6 Statistical analysis

The analysis of RSV occurrence for the different studied populations and among symptomatics and asymptomatics was made using chi‐squared, odds ratio, Fisher's exact test, Student's *t* test for independent samples, and ANOVA. The programs used were OpenEpi version 2.3.1 and GraphPad. A *P* value of <.05 was considered statistically significant.

## 3 RESULTS

We analyzed 387 samples from non‐hospitalized asymptomatic adults: 100 samples from HIV positive; 95 HCWs; 192 caregivers (all household contacts) of 196 symptomatic outpatient children. The symptomatic outpatients’ adults included were 43 HIV patients and 70 HCWs.

Of the 696 samples analyzed, 85.4% (594/696) were collected from March to September, corresponding to the period of greatest circulation of RSV in Sao Paulo city.

The characteristics of asymptomatic and symptomatic subjects are described in the Table 1.

**Table 1:** Characteristics of the studied groups

HIV	Asymptomatics (%)	Symptomatics (%)
	n = 100	n = 43
Male	69 (69.0)	29 (67.4)
Mean age (y)	46.1	43.5
Median age (y)	47.0	45.0
Comorbidities	53 (53.0)	22 (51.2)
CD4 (Cells/mm^3^)	510.5	505.8
Average time from onset of symptoms until the date of sample collection (d)	‐	8.8

Healthcare workers	n = 95	n = 70
Male	18 (18.9)	16 (22.9)
Mean age (y)	33.5	33.3
Median age (y)	32.0	28.5
Comorbidities	19 (20)	14 (20)
Average time from onset of symptoms until the date of sample collection (d)	‐	5.3

Asymptomatic contacts	n = 192	‐
Male	18 (9.4)	‐
Mean age (y)	34.8	‐
Median age (y)	34.0	
Comorbidities	36 (18.7)	‐

Children	‐	n = 196
Male	‐	102 (55.6)
Mean age (y)	‐	3.5
Median age (y)		2.0
Comorbidities	‐	17 (8.7)
Average time from onset of symptoms until the date of sample collection (d)	‐	3.14

The overall positivity found among the studied populations was 10.1% (70/696). The RSV detection rate was 4.4% (17/387) for asymptomatic individuals and 17.1% (53/309) for symptomatic individuals. (*P* < .0001). Someone infected with RSV has 4.5 greater odds of presenting symptoms [OR 4.5 (95% CI 2.5‐7.9)], than being asymptomatic. The exclusion of children patients from this analysis showed a positive rate of 5.3% (6/113) for the symptomatic and 4.4% (17/387) for the asymptomatic subjects (*P* = .62). Nonetheless, RSV positivity of asymptomatic caregivers (6.8%; 13/192) obtained a significant difference compared to other asymptomatic adults subjects individuals, HIV, and HCWs (2.0%; 4/195; *P* value = .0252].

### 3.1 HIV‐seropositive patients

In the group of asymptomatic patients, RSV was detected in 3% (3/100) of the samples. The average age of these patients was 33.7 years and the mean CD4 T‐cell count was 499 cells/mm^3^, without difference between those infected or not infected by RSV (*P* = .94). Two RSV cases reported previous contact with a symptomatic person. We quantified RSV viral load for 2 samples and obtained 1.4 and 5.3 log copies/mL.In the group of symptomatic HIV individuals, the positivity of RSV was 7% (3/43) and the average age of these positive patients was 46 years. The mean CD4 of patients with positive samples was 442.6 cells/mm^3^. There was no statistically significant difference between the CD4 T‐cell count of RSV‐positive and the RSV‐negative ones. (*P* = .72). The average time from onset of symptoms until the date of sample collection was 11 days for patients with positive samples.

Only 1 of the individuals reported close contact with symptomatic children at home.

The viral load found for each positive sample of symptomatic patients was 5.3, 6.5, and 3.2 log copies/mL, with an average value of 4.8 log copies/mL.

### 3.2 Healthcare workers

The RSV‐positive rate for asymptomatic HCWs was 1% (1/95) and 4.3% (3/70) for the symptomatic subjects. (*P* = .31).

The RSV asymptomatic case was 44 years old and reported contact with symptomatic patients at work. The RSV viral load of this sample was 3.0 log copies/mL.

The 3 RSV symptomatic cases (mean 33.3 years of age) had their samples collected in 5.3 days (mean) and 2 of them reported a previous contact with symptomatic children at home. Viral loads obtained for 2 of these 3 samples were 8.4 and 1.1 log copies/mL.There was no statistically significant difference for the positivity rates among symptomatic and asymptomatic groups comparing the populations of HCWs and HIV patients.

### 3.3 Symptomatic children

An RSV rate of 24% (47/196) was obtained for children included in the study. The mean age of children who had positive samples was 2.9 years (0.25‐12 years). The mean age of children with positive samples was not statistically different from the mean age of 3.8 years among children with RSV‐negative samples (*P* = .17). The average time from onset of symptoms until the date of sample collection was 3.4 days (range 1‐7 days).The average viral load for the samples was 4.6‐log copies/mL, ranging from 1.2 to 8.4‐log copies/mL. We found significantly higher viral load for children younger than 2 years of age compared with children older than 2 years of age (*P* = .0077). The average values ​​for viral load found in the different age groups of children are listed in Table 2.

**Table 2:** Analysis of RSV viral load in children samples according to age

Age (y)	Number of samples	VRSH Viral load (log copies/mL)		
		Mean	Median	Standard deviation
<0.5	6	6.7	6.6	1.1
≥0.5‐<1	6	5.5	5.5	1.3
≥1‐<2	7	4.3	4.5	1.9
≥2‐<3	9	4.6	4.6	1.8
≥3‐<4	1	a5.8	‐	‐
≥4‐<5	3	4.5	4.4	1.4
≥5	12	3.2	4.4	1.4

a Value related to a single positive sample found in this age group and not to an average.

### 3.4 Asymptomatic caregivers’ contacts

Respiratory syncytial virus positivity was found in 6.8% (13/192) of the samples among the group of asymptomatic caregivers. Mean age of RSV‐positive cases was 34.3 years (20‐64 years); 11 (84,6%) of them living at home with symptomatic children tested positive for RSV in this study. In this case, living with an RSV‐infected child at home was 22 times more likely to be infected with RSV [OR 22.6 (95% CI 4.8‐106.7)]. RSV viral load of the 13 samples varied from 0.1 to 5.4‐log copies/mL (mean 2.1 log copies/mL).

### 3.5 Viral load analysis among studied populations

The average viral load of samples from the symptomatic group and the asymptomatic group was 4.7 log/mL (median of 4.9 log/mL) and 2.3 log/mL (median of 1.9 log/mL), respectively. The symptomatic individuals showed higher RSV viral load than asymptomatic individuals (*P* < .0001). This difference between groups remained statistically higher even after excluding the children from the analysis of values obtained for symptomatic adults (4.9 log copies/mL and a median of 5.4 log copies/mL) and those asymptomatic (*P* = .0159).

It was not found statistically significant difference between the symptomatic adult group and the symptomatic children group in the analysis of viral load values (*P* = .82).

The average RSV viral load for the 11 samples from asymptomatic caregivers infected was 2.5‐log copies/mL and for the 11 children was 6.4‐log copies/mL. The symptomatic children have higher viral load than their asymptomatic contacts (*P* < .0001).

The RSV viral load among children with an asymptomatic contact infected with RSV obtained an average of 6.4 log copies/mL (3.9‐8.4; median 6.6 log copies/mL.). Among children without an asymptomatic contact infected with RSV, the quantification was 4.1‐log copies/mL (1.6 −6.5; median 4.1 log copies/mL). These children possibly transmitting the virus to their asymptomatic contacts had significantly higher viral load than children who probably not transmit (*P* < .0001).

**Figure 1:**
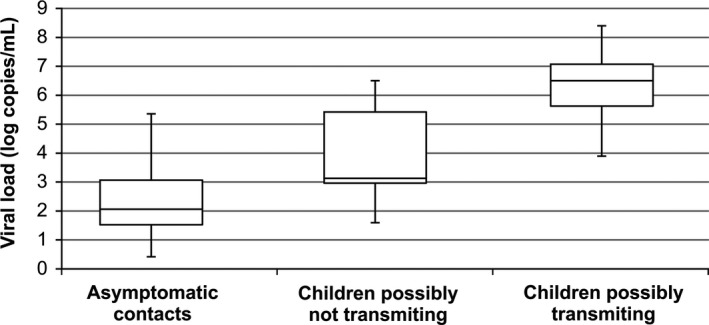
Viral load variation among the groups of asymptomatic contacts, children possibly not transmitting RSV, and children possibly transmitting RSV

Figure 1 shows viral load of RSV‐infected caregivers and children according to viral load. Viral load of children is presented in different box if possibly transmission suspected or not.

## 4 DISCUSSION

Human respiratory syncytial virus is considered an important viral agent causing upper respiratory and lower respiratory tract infection, especially in children, the elderly, and immunocompromised adults.^12^, ^13^, ^14^ Some studies have also shown the importance of RSV as a causative agent of respiratory tract infections in healthy adults, causing significant morbidity.^15^

Studies of RSV epidemiology in healthy individuals are uncommon, and the risks of RSV transmission from infected children to other individuals are barely known.^2^ The positivity for RSV among the studied populations varied within and between symptomatic and asymptomatic groups. The RSV detection data in adult patients are usually variable.^16^, ^17^

The high frequency of RSV infection in the population of asymptomatic caregivers’ contacts demonstrates the high degree of exposure to which individuals who have infected children at home are submitted. We observed that 84.6% of RSV‐positive samples in this group belonged to individuals who were asymptomatic contacts of children who had positive sample for RSV. A close contact with a child infected with RSV at home increases by 22.6 times the chance of acquiring the virus. The close contact with RSV‐infected children at home could be an important factor to the increase in the risk of acquiring RSV infection. In a recently published study, researchers detected RSV in 47% of family members of children hospitalized for infection by the virus and 82% of the detected episodes were related to the presence of respiratory symptoms.^18^

Healthcare workers are expected to be more exposed to infectious agents than community population. In the group of healthcare workers, detection was below the average found in the literature for adults11. One possible reason for that is that the HCWs’ intensive program for infection control launched at the university hospital may have led to an increase in hand‐washing practices. Another point is that samples were collected in various hospital wards in order not to concentrate in pediatric sectors. It is interesting to notice that 3 of the 4 healthcare workers with positive sample for RSV reported close contact with children younger than 5 years of age at home.Published data about the RSV infection among HIV‐positive patients are scarce, except for some case reports. In our study, HIV patients presented high level of CD4 count so that susceptibility to community respiratory viral infections is not different for common population. The average time from onset of symptoms until the date of sample collection in infected patients from this group was high (11 days), and additional studies related to RSV infection among these patients would understand the dynamic of this type of viral infection.

New drugs have been studied in challenge trials and therapeutically reduced both viral load and clinical manifestations of RSV infections.^19^ In this regard, studies including viral load are mandatory to support the use of new drugs in the future when available for clinical use.We observed that the viral load of symptomatic patients has significantly higher value than the viral load of asymptomatic patients and the viral load has no significant difference between adults and children. These results may reflect the need for highest viral replication rates to cause symptoms. High levels of viral load in adults’ samples are usually associated to more ​​severe symptoms, complications, and hospital admissions.^20^, ^21^

In the analysis of the viral load in asymptomatic caregivers’ contacts of symptomatic children, the viral load in children's samples was significantly higher than the viral load in their contacts and significantly higher than the viral load of children without an infected caregiver. This may indicate a need for high viral load values to occur the transmission.

Although considering the limitations of our cross‐sectional study, mainly the absence of following the patients and the non‐differentiation of species A and B, it was demonstrated that the close contact with infected children is an important risk factor for acquiring RSV infection. This risk could be extended to healthcare workers, HIV‐positive patients, and visitors of hospitalized patients who have contact with children at home mainly during RSV seasons.Additional studies should be conducted for a better understanding of the clinical significance of asymptomatic RSV infection carriers on nosocomial transmissions during RSV seasons.

According to our results, it is important to know if people circulating inside the hospital have close contact with acute respiratory infected children. Indeed, the need to maintain the team of professionals and the public informed about the infection prevention and control measures against the acquisition of infection by respiratory syncytial virus is critical.
